# Mark Pitkin “Practical Sanomechanics®: Exercising for a Healthy Skeleton”

**DOI:** 10.2478/hukin-2014-0082

**Published:** 2014-10-10

**Authors:** Maxim Shevtsov

**Affiliations:** 1 Institute of Cytology of the Russian Academy of Sciences (RAS), St. Petersburg, Russia.

**Keywords:** sanomechanics, floating skeleton theory, rehabilitation

Part I - Method of Sanomechanics - contains two chapters. The first, Sanomechanics Foundation, describes the author’s theory of the distribution of pressures between the joints’ capsules. Sanomechanical Approach to the Risks of Routine Motions, which follows, applies that theory. The main idea is that once a joint is loaded, pressure exerted on cartilage is transmitted hydrostatically to other joint capsules. Morphologically, the pressure is transmitted through tiny spaces between the bone and the periosteum sheath that covers the entire skeleton. While it is a challenging hypothesis, it is quite appealing in how it logically suggests a remedy for overloading of joints and a method for rehabilitation in case of joint pain: the hydrostatic connectivity has to be restored, and the skeleton has to “float” again in the subperiosteal film of fluid.

Part II - Practical Sanomechanics – teaches the reader how to maintain -- and restore if needed -- effective functioning of the floating skeleton. In the chapter called Sanomechanical Technique, the reader will find clearly written instructions for exercising in a sanomechanical fashion. Sanomechanics Exercises gives well illustrated examples of a routine that applies to different parts of the body and to different situations in a daily life.

The term sanomechanics is derived from Latin and it represents the science of health as a subject of forces generated by motion of the body. Professor Pitkin presents a well justified system of exercises which differs in principle from all known systems of perfecting the body. Often we may find exercises to be painful or uncomfortable. What should we do to recuperate without medication?

A viewer watching how one does the sanomechanics routine, may not distinguish it from yoga, tai chi, stretching, etc. However, the reader of this book will immediately learn that the sanomechanics practitioner takes the body positions and the postures being developed over the centuries and enhance them with the three original conceptual discoveries.

The first one is the floating skeleton theory, stating that the synovial capsules form a hydraulic net enable to transmit the excessive pressures from the overloaded joints. That system is an effective protector for the contacting cartilages. When and if the hydraulic connection is broken and the joint is hydraulically isolated from the net, the cartilages become overloaded and eventually damaged.

So, the purpose of the sanomechanical exercises is to maintain and restore the skeleton as a structure “floating” inside the periosteum.

In order to reach this newly proclaimed morphological and physiological system in the body, the author suggests a criterion of correctness of keeping the postures. That criterion is called the Hedonic (pleasure) principle, routed in the “pleasure principle” introduced by Freud.

The third component of the sanomechanics system is inclusion of autosuggestion, which originated in ancient meditation techniques.

A person equipped with these components, as I found for myself, can effectively and surprisingly quickly feel the benefits of sanomechanical exercises aimed at prolonging the health of our skeleton and the whole body.

The book is a must for all health and physical activity specialists. It is novel, highly informative and practical.

